# Assessment of Alginate Gel Films as the Orodispersible Dosage Form for Meloxicam

**DOI:** 10.3390/gels10060379

**Published:** 2024-06-02

**Authors:** Barbara Jadach, Martyna Kowalczyk, Anna Froelich

**Affiliations:** 1 Division of Industrial Pharmacy, Chair and Department of Pharmaceutical Technology, Poznan University of Medical Sciences, 3 Rokietnicka Street, 60-806 Poznań, Poland; martyna4.kowalczyk@gmail.com; 2 3D Printing Division, Chair and Department of Pharmaceutical Technology, Poznan University of Medical Sciences, 3 Rokietnicka Street, 60-806 Poznań, Poland

**Keywords:** orodispersible gel film, fast disintegration, alginate gel film, meloxicam

## Abstract

The aim of this study was to obtain films based on sodium alginate (SA) for disintegration in the oral cavity. The films were prepared with a solvent-casting method, and meloxicam (MLX) as the active ingredient was suspended in a 3% sodium alginate solution. Two different solid-dosage-form additives containing different disintegrating agents, i.e., VIVAPUR 112^®^ (MCC; JRS Pharma, Rosenberg, Germany) and Prosolve EASYtabs SP^®^ (MIX; JRS Pharma, Rosenberg, Germany), were used, and four different combinations of drying time and temperature were tested. The influence of the used disintegrant on the properties of the ODFs (orodispersible films) was investigated. The obtained films were studied for their appearance, elasticity, mass uniformity, water content, meloxicam content and, finally, disintegration time, which was studied using two different methods. The films obtained with the solvent-casting method were flexible and homogeneous in terms of MLX content. Elasticity was slightly better when MIX was used as a disintegrating agent. However, these samples also revealed worse uniformity and mechanical durability. It was concluded that the best properties of the films were achieved using the mildest drying conditions. The type of the disintegrating agent had no effect on the amount of water remaining in the film after drying. The water content depended on the drying conditions. The disintegration time was not affected by the disintegrant type, but some differences were observed when various drying conditions were applied. However, regardless of the formulation type and manufacturing conditions, the analyzed films could not be classified as fast disintegrating films, as the disintegration time exceeded 30 s in all of the tested formulations.

## 1. Introduction

The oral route of drug administration is the most acceptable to patients [1]; however, some oral forms may cause inconveniences, especially in patients with swallowing difficulties [2]. To avoid the risk of choking, the active substance can be administered in the form of a film quickly disintegrating in the oral cavity [3]. This approach may be considered as advantageous from many points of view. First of all, it makes it much easier to use in pediatric or geriatric patients, because disintegration occurs within 30 s [4]. An additional portion of liquid is not necessary, and no solid fragments are left in the oral cavity [1,5]. Compared to liquid formulations, there is no risk of inaccuracy in measuring the dose, and, at the same time, the dose personalization is quite simple. Quickly disintegrating films contain an active pharmaceutical ingredient (API), film-forming polymer, disintegrants and other excipients [3]. Orodispersible dosage forms have been gaining growing popularity in the pharmaceutical market since 2003, mostly as easy-to-use formulations with minimized risk of choking [5,6]. According to the European Medicines Agency (EMA), orodispersible films (ODFs) do not melt but they dissolve or disintegrate upon contact with saliva. Another dosage form with similar features is orally disintegrating tablets (ODTs). In both forms, quick disintegration is an important advantage, and, in addition, ODFs leave smaller amount of solid residue in the mouth after disintegration compared to ODTs, which further minimizes the choking risk [1,6,7,8]. As was already mentioned, an ODF is a thin flexible layer composed mainly of polymer and active substance but may also contain plasticizers and salivation stimulants, fillers and substances that improve appearance and taste, in the form of dyes, sweeteners or substances masking the taste and smell of the API [1,3,7,9]. The perfect film should be thin and flexible but durable enough to avoid damage during production, packaging, storage and application. Moreover, it should not be sticky, and its sensory properties, including taste and feel in the mouth, are also important [7,8]. The matrix disintegration time should be as short as possible; however, better mechanical properties of the film are often associated with an extended disintegration time [1,3,7]. Thin polymer films can be prepared using various methods, and, among the most frequently described ones, solvent-casting [3,6,10,11], hot-melt extrusion [12,13,14], 3D printing and electrospinning [13,15,16] techniques should be mentioned.

**Figure 1:**
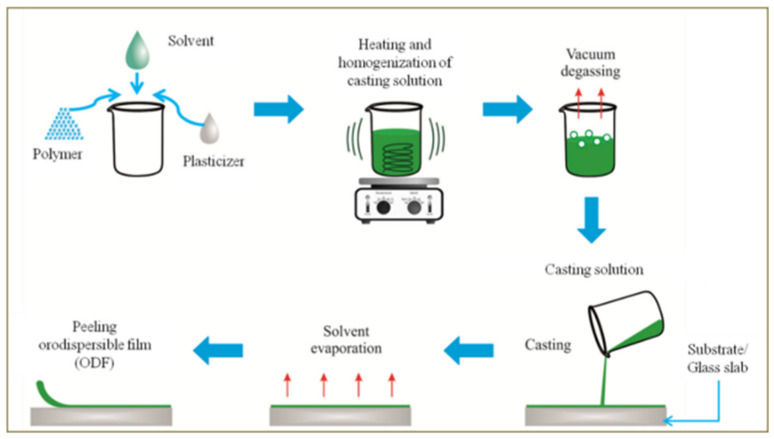
Solvent casting method (reprinted from [10]).

The solvent evaporation method, known also as solvent casting (Figure 1), is frequently employed in the production of polymer films due to its simplicity and low process costs [3,6,10,17,18]. The first step of the process is the selection of a solvent for the polymer and the active substance. Next, the polymer solution is prepared and its viscosity and the temperature during mixing should be optimized. Any air bubbles introduced into the mixture during mixing must be removed before pouring the mixture, to obtain a homogeneous product. When the obtained polymer mass is homogeneous, the active substance and additives can be added, ensuring even distribution of the drug in the film. Rheological properties at this stage are an important control parameter that may affect the drying process, film thickness or its morphology. Next, a thin layer of the prepared mixture is poured onto a specially prepared surface and dried with hot air. The drying temperature, time and air humidity should be controlled. After drying, the obtained polymer film is cut into pieces of appropriate mass and API dose and packed [3,6,10].

The hot-melt extrusion method involves mixing the dry ingredients, including API, drug, plasticizer and other excipients, melting the mixture and homogenizing it without the use of a solvent [12,14,19]. However, this method is unsuitable for thermolabile substances due to the relatively high temperatures during the process [3,19]. Other film manufacturing methods like printing, two-dimensional inkjet printing, 3D-printing and flexographic printing [10,13] are interesting because they can be used to obtain single-layer or multi-layer films with different physical and mechanical properties of the obtained product [20].

In the presented work, sodium alginate (SA), a natural polysaccharide, was used as a film-forming polymer [21,22,23]. Alginates are composed of α-L-guluronic acid (G) and β-D-mannuronic acid (M) units connected by a β-1,4-glycosidic bond. They are organized into sequences of homogeneous blocks (GG, MM) or heterogeneous blocks (GM) [21,22,24]. Alginate is used in many industries, including food technology, mainly as a thickener and suspensions and emulsions stabilizer [22]. Due to its high versatility and good biocompatibility, the polymer has also numerous biomedical and pharmaceutical applications [25,26,27]. It can be employed in the manufacturing of various liquid, semi-solid or solid forms and is particularly useful in formulating emulsions, gels and suspensions [24]. Because of its excellent swelling properties, it can be applied as a component of delayed or prolonged release systems [26,27,28]. SA has mucoadhesive properties, so it is a suitable polymer for creating buccal, intranasal and ophthalmic formulations [21,29], as well as vaginal dosage forms [30,31]. Also, alginate-based dressings applied to a wound with exudate facilitate the healing process and growth of fresh epidermis by maintaining a physiologically moist environment [32,33]. Alginates are also used as structural biomaterials supporting the reconstruction of tissues, e.g., teeth, bones, cartilage [21,34,35,36].

Meloxicam (MLX) was selected as the API, belonging to the group of non-steroidal anti-inflammatory drugs (NSAIDs) and acting as a selective cyclooxygenase-2 inhibitor. MLX has analgesic, anti-inflammatory and antipyretic effects and is mainly used to treat the pain associated with rheumatic diseases in children and adults [37] and is widely used in therapy [37,38,39,40,41,42].

The aim of the presented research was to obtain and evaluate the physicochemical properties of alginate-based films containing two different additives for solid dosage VIVAPUR 112^®^ (MCC) and Prosolve EASYtab SP^®^ (MIX). The first one contains microcrystalline cellulose only, while the other one is a mixture of different solid dosage forms components, also including sodium starch glycolate as a disintegrating agent. The most important objective of this study was to assess the impact of these additives on the film disintegration time. Moreover, the influence of the drying conditions on the properties of the final product was analyzed.

## 2. Results and Discussion

Polymer gel films are oral formulations that are less popular than tablets or liquid dosage forms [1,3,43]. In the presented studies, sodium alginate was used as the film former [17,18], with two compositions proposed for the film preparation (Section 4.2). Similarly to our previous study [44], the solvent casting method was successfully used for the preparation of alginate films. Both proposed compositions were dried with the use of four condition combinations, i.e., 30 °C, 48 h (A); 30 °C, 72 h (B); 40 °C, 24 h (C) and 50 °C, 24 h (D). At the beginning, the films were dried at 30 °C, for 24 h, and they were too wet. It was not possible to take them off from the plates, so, in the next step, the time of drying was extended. However, the plan was to dry the material in a shorter time, and, for this purpose, the temperature was increased. At 48 h of drying at 40 °C or 50 °C, the films cracked, so the decision was made to shorten the time to 24 h for the higher used temperatures.

### 2.1. Amount of MLX

The meloxicam content in all batches remains approximately the same (Table 1).

The highest content was observed for the C-MCC series, and the highest variability within one batch was reflected by the highest standard deviation for the D-MIX series. Small deviations in the results obtained in this study may confirm the homogeneous distribution of solid particles of the drug substance in the mass prepared for pouring films onto plates. With the proposed formulations, it would be possible to obtain a single, individual dose for the patient by preparing a film of a specific size.

### 2.2. Appearance of Gel Films

**Figure 2:**
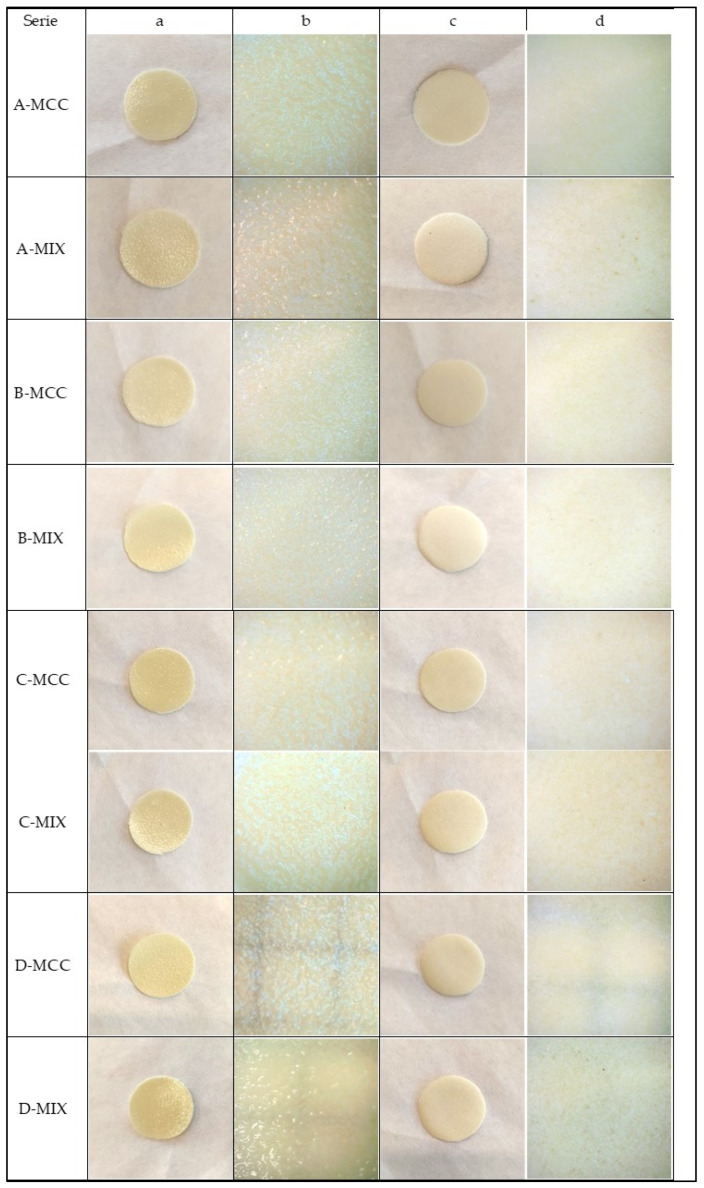
Comparison of the appearance of the films taken with Inskam 316 microscope; (**a**,**b**) top surface; (**c**,**d**) bottom surface.

The appearance and main characteristic of each prepared batch of polymer films is presented in Figure 2, and the characteristics are collected in Table 2. When visually assessing the prepared films, it was noticed that the upper surface of the films was rough and shiny, while the lower surface was smooth and matte. The differences in appearance are summarized in Table 2. During drying, some films cracked and curled, depending on the conditions and the type of disintegrant. However, the films were still flexible and were subjected to further examination.

One of the objectives of this study was the evaluation of the impact of drying process conditions. First, drying at 30 °C for 24 h was carried out. As it turned out, such conditions were not sufficient to obtain films that could be removed from the Petri dish and analyzed. Therefore, a longer drying time or higher temperature seemed necessary. The application of the conditions A (30 °C, 48 h), B (30 °C, 72 h), C (40 °C, 24 h) and D (50 °C, 24 h) resulted in films that could be further evaluated. It was noticed that films with the addition of the MIX additive were generally slightly more flexible but were not as uniform as those with MCC. It was found that the visually assessed elasticity decreased with the decrease in water content. The roughness shown on the upper surface appeared regardless of the drying temperature and composition. However, the bottom side adhering to the glass was always smooth. Surface roughness may result from uneven water evaporation [43]. It seems likely that the cracks in the films could have resulted from the water evaporating too quickly, using too high drying temperature or because the drying time was too long, resulting in insufficient water content in the final product. It was also noticed that cracks appeared more often when the MIX disintegrant was used.

### 2.3. Mass Uniformity of the Gel Films

**Figure 3:**
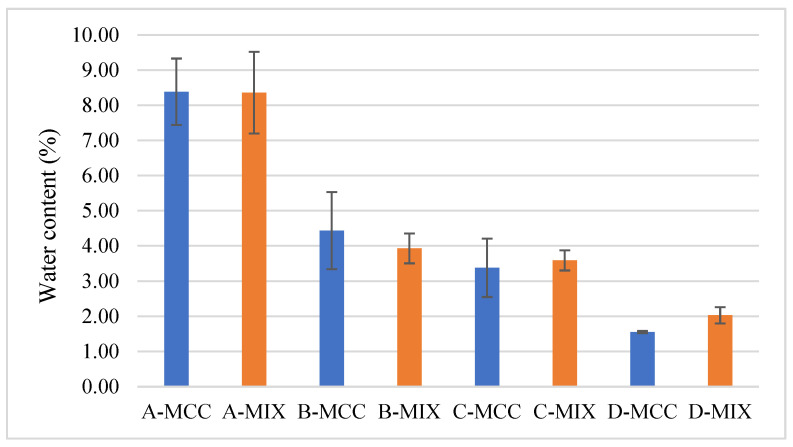
Comparison of water content in prepared films.

The results of the mass uniformity examination of the films after cutting are presented in Table 1. The C-MCC series had the highest average mass, with a relatively large standard deviation. The highest variability is observed for the D-MIX series, while the B-MIX series has the smallest weight. In general, it was noticed that for all formulations, similar film masses were obtained. The only slight differences may be related to the amount of water content in them (Figure 3). For individual drying conditions, between the series containing the MCC or MIX disintegrant, the differences in mass are very small; therefore, it can be concluded that the type of substance used did not affect the final mass and the retention of water in the film structure during the drying process.

### 2.4. Water Content

There are no regulatory requirements concerning the water content of oral films; however, the moisture contained in the films can have a significant impact on the adhesive properties and mechanical strength of the final product [45]. The highest content of water was observed in both series dried at 30 °C for 48 h. However, the water content in films dried at 40 °C for 24 h does not differ significantly from that of films dried for 72 h at 30 °C. In Figure 3, the comparison of the water content in the individual batches is presented. The lowest water content was found for the D-MCC series. A significant impact of the drying conditions on the water content in the prepared films can be observed. It seems obvious that increasing the temperature ensures drier films, but it is worth noting that at higher temperatures more uniform results (lower standard deviations) were also obtained, which may indicate a uniform drying process under these conditions. At the same time, no significant difference in water content was noticed between films dried in the same conditions and containing different disintegrating substances.

### 2.5. Disintegration Time

A study of the disintegration time was carried out by placing the film in a Petri dish with 10 mL of artificial saliva (pH 6.8), and the film was stirred with a 4 cm long magnetic stirrer, which was moved to imitate the physiological conditions in the mouth, including mechanical forces related to the movement of the tongue. There are no clear pharmacopeia requirements for a maximum acceptable disintegration time for an ODF. However, the FDA requirements and USP guidelines for ODTs are often used for polymer films. The maximum disintegration time for an ODT is 3 min [6,7,10].

**Figure 4:**
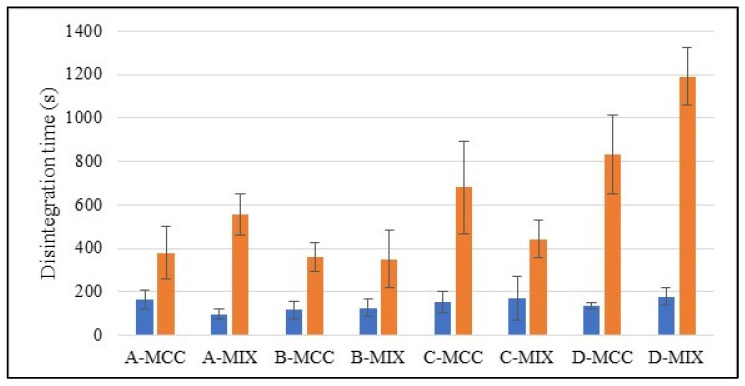
Disintegration time for all series and both methods (blue-method I, orange-method II).

With the first study method, it was observed that at the end of this study there was significant turbidity of the fluid at the end point shown. The highest disintegration time was recorded for the D-MIX batch, and the quickest disintegration was observed for the A-MIX batch (Figure 4).

**Figure 5:**
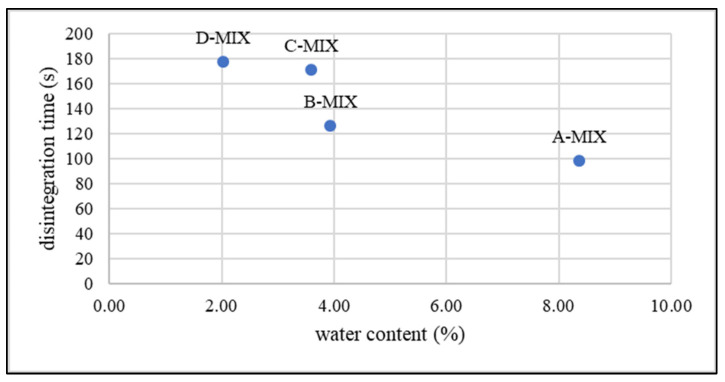
Plot of disintegration time (method I) vs. water content for series MIX.

The films with MCC prepared with different conditions seemed to be more similar to each other than the batches with MIX. Also, for the MIX series, a correlation between the amount of water and disintegration time was observed (Figure 5). For the B-MIX and C-MIX series, which were dried at 30 and 40 °C, respectively, the water content was similar (0.5% difference). The disintegration times were different, even when a similar amount of water in the film was observed. The observed differences may be related to different water diffusion rates in the polymer matrices. Moreover, for series D and C (prepared at 50 and 40 °C, respectively), which showed a difference of 1.5% in water content, the disintegration time was very similar. In the second method, a high variability in the results was observed. This could be explained by the difficulties in determining the end point of the disintegration.

According to Figure 4, the disintegration times observed in method II were longer than in method I, which also involved the mechanical forces provided by the magnetic bar. Batches A-MIX, B-MIX and C-MIX did not differ significantly. For the MCC composition, the A-MCC and B-MCC batches were similar, while the C-MCC and D-MCC batches had longer disintegration times, which increased when the drying process was performed at a higher temperature. Taking into consideration the experimental setup applied in method II, it may be assumed that the test is more static than method I, and it can reflect the diffusion of water in the polymer matrix without additional erosion caused by stirring [46]. The test showed that the materials with lower water content required more time to absorb water and initiate the disintegrants’ swelling and matrix disintegration. In the case of mechanical action simulating the movement of the tongue (method I), all the prepared films, regardless of the drying temperature used, actually behaved very similarly. In all cases, the samples disintegrated in less than 3 min. However, according to the literature, this time is too long to classify films as fast-dissolving dosage forms. The disintegration of each series of films was longer than 30 s but not greater than 180 s, which is the maximum disintegration time for orally disintegrating dosage forms according to the FDA and the US Pharmacopoeia. The proposed films were prepared from alginate solution by solvent casting, so no cross-linking agent was added. This suggests that disintegration can be due to swelling of the polymer in artificial saliva. The time was long so it could suggest that solid particles present in the structure of the film make it difficult to penetrate the polymer film [46] with artificial saliva.

## 3. Conclusions

Analyzing the results obtained during the evaluation of the proposed method of films preparation, it can be concluded that solvent casting method can be successfully used to easily prepare sodium alginate films with good elasticity. The prepared films contained a similar amount of MLX, which indicates a good repeatability of the manufacturing process. Also, the type of disintegrant used affected the appearance and elasticity of the films but did not significantly affect the disintegration time. The drying conditions applied in the film manufacturing process had a significant impact on their water content, which also partially affected the disintegration time. The range in the prepared films was quite wide from 1.56 to 8.39%; however, there are no regulations concerned with water content for this type of dosage form, although the disintegration of films depends primarily on the water content in the film, regardless of the complexity of the film formulation and the use of a specific disintegrant. The main point for the preparation of fast dissolving formulations should be the composition which leads to the acceleration of disintegration time.

## 4. Materials and Methods

### 4.1. Materials

Meloxicam (MLX) was kindly donated free of charge by Biofarm (Poznań, Poland) and was used as received. Sodium alginate was purchased from Sigma-Aldrich (St. Louis, MO, USA). Glycerol 85% was purchased from Fagron (Rotterdam, The Netherlands). MCC-VIVAPUR 112 and PROSOLVE EASYtab SP were purchased from JRS Pharma (Rosenberg, Germany). Sodium hydroxide, sodium chloride, potassium dihydrogen phosphate and potassium hydroxide were purchased from Avantor Performance Materials S.A. (Gliwice, Poland). Disodium bicarbonate and orthophosphoric acid were purchased from Chempur (Piekary Śląskie, Poland).

### 4.2. Preparation of Polymer Gel Films

Polymer films were prepared by the solvent casting method as described in our previous study [44]. Two different compositions (Table 3) containing MCC-VIVAPUR 112 (MCC series) and PROSOLVE EASYtabs SP (MIX series) were prepared and dried in each condition combination. Next, round films with a diameter of 1.9 cm were cut out and packed for storing in aluminum foil.

The films were prepared from a 3% alginate solution. For this purpose, 3 g of sodium alginate was dissolved in water with the addition of 5 g of glycerin, heated to 60 °C and stirred with a magnetic stirrer. In this way, 100 g of solution was obtained. Part of the prepared solution was combined in the evaporator dish with 0.5 g of meloxicam and 2 g of the appropriate disintegrant to obtain a homogeneous suspension. Then, the remaining amount of alginate solution was added and thoroughly mixed. The prepared dispersions were poured into Petri dishes, 25 g each. The prepared film samples were dried in different conditions: 30 °C, 48 h (A); 30 °C, 72 h (B); 40 °C, 24 h (C) and 50 °C, 24 h (D).

### 4.3. Amount of MLX in Gel Films

**Figure 6:**
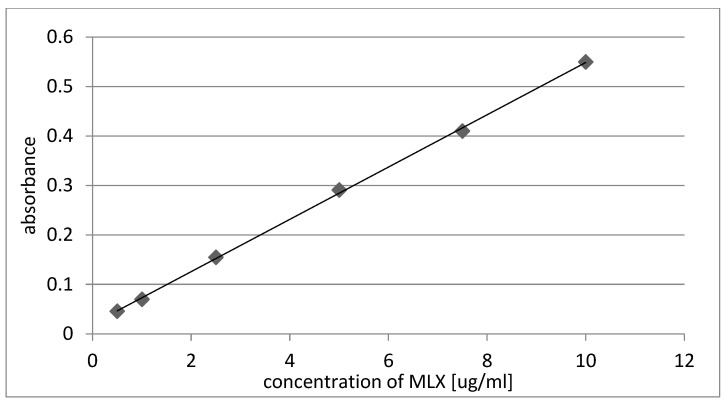
Calibration curve for determination of MLX with UV-VIS spectrophotometry.

Three samples of films from each series were weighed and each of them was dissolved in 50 mL of NaOH (0.1 M). Then, the solutions were filtered with 0.22 μm membrane filters, and 1.5 mL of each solution was diluted to 20 mL. The amount of MLX was measured with UV–VIS spectrophotometry, and the recorded absorbance values were used to obtain MLX concentrations with the use of validated calibration curve (Figure 6), equation: y = 0.0528x + 0.0203; coefficient r^2^ = 0.9995.

### 4.4. Appearance, Size and Mass Uniformity of the Gel Films

In each of the investigated batches, 10 films were randomly selected and weighed to determine mass uniformity. Average mass values, standard deviations and coefficients of variation for each batch were calculated. Observations were also made regarding the surface structure of the films. Films were observed under an Inskam 316 microscope (Dongguan, China). To determine elasticity, a film from each series was bent to see if a fracture would occur in this place.

### 4.5. Water Content

Similarly to our previous study [44], the water content in the prepared films was determined using the Karl Fisher titration method performed with a Karl Fisher Mettler Toledo (Columbus, OH, USA) titrator. The measurement was carried out with the volumetric method involving an iodine solution, two-component Titrant 5 (Aquastar, Merck Darmstadt, Darmstadt, Germany) and methanol as a solvent. The measurement was started by filling the titration cell with solvent to a volume of 40 mL and, for each batch, was performed in triplicate (n = 3). The water content was calculated based on the volume of the added titrant in the tested sample.

### 4.6. Disintegration Time of Gel Films

In order to determine the disintegration time of the films, two methods involving the use of artificial saliva were employed. Method I was prepared according to Scarpa et al. [43] with a slight modification.

**Figure 7:**
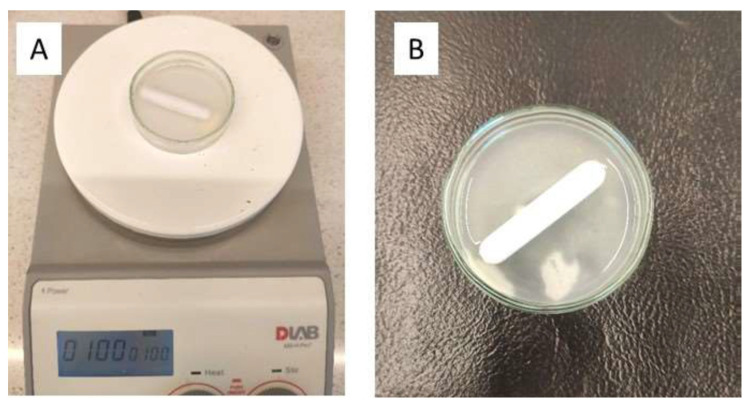
Examination of disintegration time with method I (**A**); the endpoint of the investigation (**B**).

Film samples were placed in 10 mL of artificial saliva at 37 °C in a Petri dish, and the temperature was kept constant throughout this study. The contents were mixed using a magnetic stirrer (4 cm) at 100 rpm to imitate the mechanical friction occurring in the oral cavity (Figure 7A). The endpoint (Figure 7B) was defined as the time after which the film broke into smaller pieces or the moment when half of the film dissolved.

**Figure 8:**
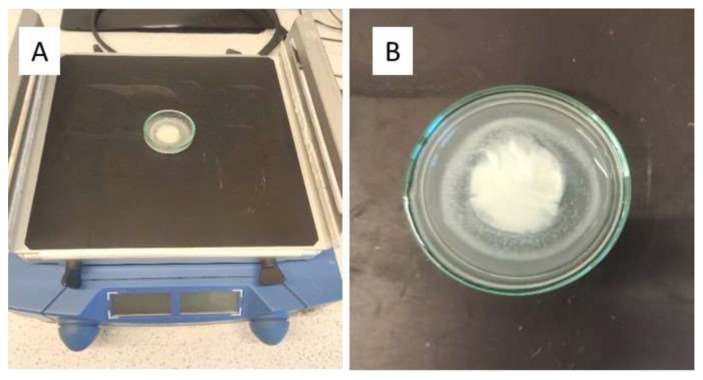
Examination of disintegration time with method II (**A**); the endpoint of the investigation (**B**).

In method II, the disintegration was carried out in 2 mL of artificial saliva, also on Petri dishes subjected to shaking at 150 rpm (Figure 8A) [8,46].

The initial temperature of the liquid was 37 °C, but the shaker was kept at ambient temperature to visually monitor the study endpoint (Figure 8B).

## Figures and Tables

**Table 1:** Characteristics of gel films.

Series	Mass Uniformity (g)	Amount of MLX (mg)
A-MCC	0.124 ± 0.014	5.8 ± 0.4
A-MIX	0.129 ± 0.015	4.7 ± 0.4
B-MCC	0.119 ± 0.013	4.9 ± 0.3
B-MIX	0.114 ± 0.013	4.7 ± 0.4
C-MCC	0.134 ± 0.015	6.3 ± 0.3
C-MIX	0.128 ± 0.010	5.3 ± 0.1
D-MCC	0.123 ± 0.011	5.7 ± 0.5
D-MIX	0.124 ± 0.017	6.0 ± 1.0

**Table 2:** Characterization of the appearance of the films.

Serie	Cracking	Bending	Homogenity
A-MCC	did not crack	very slight breakage or non-existent	yes
A-MIX	cracked and curled slightly, but remained very flexible	no fracture visible and the film does not constantly deform	on the bottom side slight heterogeneity
B-MCC	only slightly cracked	slight break and deformation occurs	yes
B-MIX	cracked more than the B-MCC series	slight deformation when bent without any fractures	yes
C-MCC	cracked slightly	slight break when bending, but in general the films are flexible	yes
C-MIX	clearly cracked	no breakage but there is a slight deformation	heterogeneity on the bottom surface
D-MCC	visibly cracked and curled	fracture and deformation remain on the bent film	yes
D-MIX	visibly cracked and curled	fracture and deformation remain on the bent film	yes

**Table 3:** Composition of gel films.

Ingredient *	ALG-MCC	ALG-MIX
	Weight (g)	
Sodium alginate	3.0	3.0
PROSOLVE EASYtab SP	-	2.0
VIVAPUR 112 (microcrystaline cellulose)	2.0	2.0
Meloxicam	0.5	0.5
Glycerol	5.0	5.0

* Composition for 100 g of gel film mass.

## Data Availability

The original contributions presented in the study are included in the article.
